# High-quality cooperative research: studies that represent a triumph in the rheumatology community

**DOI:** 10.1186/s13075-019-2024-6

**Published:** 2019-11-11

**Authors:** Teresa A. Simon

**Affiliations:** Physicians Research Center, LLC, Toms River, NJ 08753 USA

**Keywords:** Editorial, Meta-analysis, Rheumatoid arthritis, Biologics, Safety, Real-world research

## Abstract

Over the past 20 years, the rheumatoid arthritis (RA) treatment landscape has been continuously evolving. A range of novel biologic agents, different from the conventional therapies, became available. However, some understandable concerns, such as long-term safety, accompanied their development. Over the years in rheumatology research, I aimed to broaden the knowledge of the new treatments of RA through real-word research, which proved to be valuable in providing important evidence to clinicians and enabling them to make informed treatment decisions. Nevertheless, many unanswered questions remain—it will be interesting to see how the research progresses over the next 20 years.

My career in rheumatology began 25 years ago. Early on, I met a close-knit group of rheumatologists and epidemiologists; we worked and learned together over the years to increase our understanding of patients with rheumatoid arthritis (RA), their disease, and the available treatments. In the 1990s, breakthrough antirheumatic therapies were emerging. The standard of care was methotrexate, a disease-modifying antirheumatic drug (DMARD). The new biologic DMARDs (tumor necrosis factor inhibitors) were designed to reduce inflammation and possibly stop disease progression; however, there was a concern about their long-term safety. Regulatory authorities imposed mandatory monitoring of the safety (specifically, development of malignancies) of these medications for ≥ 5 years [1]. A decade later, the development of a new biologic—abatacept—for RA treatment was initiated. Abatacept has a distinct mechanism of action; it does not block inflammatory proteins like tumor necrosis factor-alpha antagonists, but attaches to the surface of inflammatory cells and blocks a specific interaction between them. Through this blocking, abatacept lessens inflammation and decelerates disease progression [2]. I found myself intimately involved with the development of this new therapy.

As a new rheumatology researcher, I needed to better understand the scientific issues and regulatory expectations around treatment. What was our understanding of RA, its co-morbidities, available treatments, and their side effects? What were the unmet needs, knowledge gaps, and areas requiring further clarification? We knew that patients with RA were mostly female with a history of smoking and an increased risk of developing lymphoma [3, 4]. It was uncertain whether this increase was independent of or associated with RA treatments. Thus, treating physicians were cautious regarding the new biologics and possible cancer development. Interestingly, studies assessing the occurrence of malignancies associated with biologics used the general population (GP) as a comparator (specifically US package inserts and product labels). Thus, unanswered questions remained. What was the background rate of malignancies in patients with RA? In addition to lymphoma, was the occurrence of malignancies in patients with RA different from the GP? For most RA cohort studies, the local GP was used as the comparator. What could meta-analyses of these data reveal? This question led to our extensive literature meta-analysis publication in 2008 [5], showing no difference in overall malignancy rates between the RA population and GP, with some differences for certain cancers. Lymphoma and lung cancer incidences were higher in the RA population versus the GP, but colorectal and breast cancer incidences were lower. These observations, except for lymphoma, were new, suggesting that patients with RA had different background rates of specific malignancies versus the GP and that an untreated patient with RA may be the most appropriate comparator for biologic studies.

I was fortunate to work for a company interested in understanding the truth and that provided me the opportunity to establish a program to measure the long-term risks associated with abatacept. This support enabled me to consult with renowned epidemiologists and rheumatologists across the world. Together, we built a team that answered central questions in rheumatology and cancer, implementing a large real-world study to understand the background rates of malignancies and infections in patients with RA treated with conventional DMARDs [6]. These data were pivotal in advancing rheumatology research.

Abatacept had a 10-year post-marketing commitment; monitoring the occurrence of malignancies was one of its objectives. To meet this requirement, the same group of rheumatologists with some new fellows (a consortium of sorts) developed a larger program to monitor safety of biologics in the real-world setting. The program consisted of updating the previously published systematic literature search [7] data from ten registries (in North America and Europe), four US claims databases, and a pregnancy registry [8]. This program has since completed; some results were presented in 2019 [8]. Data showed that abatacept patients were similar with respect to age and prior biologic exposure across all sources. Many patients (44–85%) had prior exposure to ≥ 2 biologics relative to other biologic and targeted synthetic DMARDs (0–19%). Does that make them different? Most likely. This now becomes the challenge for emerging new agents. What is the most appropriate comparator? With many treatments available, patients are prescribed a biologic early in their disease or may cycle through biologics more quickly, making it challenging to identify a “good” RA comparison group. New epidemiologic study designs, e.g., the prevalent new-user design with time-conditional propensity scores, were developed to address these issues and have been applied in the context of abatacept safety in patients with RA and chronic obstructive pulmonary disease [9].

There remains a need for real-world evidence in antirheumatic drug development due to the complexity of the disease and its treatments. Understanding the disease, its signs, symptoms, and unmet treatment need is critical. With the availability of robust data, researchers need to continually review evidence that is generated to make informed decisions and build solid research programs.

Per Henri Poincaré, “science is built on facts, as a house is with stones. But a collection of facts is no more science than a heap of stones is a house” [10]. A century later, we may paraphrase this quote, replacing the elusive term “facts” with concrete “data.” We seem consumed by an overabundance of information, much of which is neither robust nor useful.

As a researcher, it is important to learn and understand the population of interest by applying all forms of methodology (quantitative, qualitative, and mixed), with an aim to uncover the “truth.” As researchers, we also look for patterns and associations by using information from “good” research. I have applied these tools for the past 30 years to learn about the differences between patients who are prescribed a medication and those who are not. I continually strive to understand the background or noise. What is new or different? How have patients changed?

One of the addressable problems at the forefront is a need to identify robust data. The effectiveness of sophisticated epidemiological methods can be lessened by arbitrary data collection strategies. It will be interesting to see where we are in 20 years.

## Data Availability

Not applicable

## Abbreviations

DMARD: Disease-modifying antirheumatic drug
GP: General population
RA: Rheumatoid arthritis
